# Bidirectional Relationship between Chronic Kidney Disease & Periodontal Disease

**DOI:** 10.12669/pjms.291.2926

**Published:** 2013

**Authors:** Arsalan Wahid, Saima Chaudhry, Afifa Ehsan, Sidra Butt, Ayyaz Ali Khan

**Affiliations:** 1 Arsalan Wahid, M. Phil Scholar, Department of Oral Health Sciences, Shaikh Zayed Medical Complex, Lahore, Pakistan.; 2 Saima Chaudhry, PhD, Lecturer (Oral Pathology),Department of Oral Health Sciences, Shaikh Zayed Medical Complex, Lahore, Pakistan.; 3 Afifa Ehsan, M. Phil Scholar, Department of Oral Health Sciences, Shaikh Zayed Medical Complex, Lahore, Pakistan.; 4 Sidra Butt, M. Phil Scholar, Department of Oral Health Sciences, Shaikh Zayed Medical Complex, Lahore, Pakistan.; 5 Ayyaz Ali Khan, PhD, Head (Community Dentistry), Department of Oral Health Sciences, Shaikh Zayed Medical Complex, Lahore, Pakistan.

**Keywords:** Periodontitis, Hemodialysis, Chronic kidney disease, Albumin, C-reactive protein

## Abstract

Non communicable diseases (NCDs) affect the life of an individual in terms of mortality, morbidity and financial crises. Main NCDs are diabetes mellitus (DM), cardiovascular diseases (CVD), pulmonary diseases, osteoporosis and chronic kidney diseases (CKD). About 40% of the total deaths can be controlled by eliminating the risk factors for NCDs. Periodontitis have recently been labeled as an important potential risk factor for NCDs. CKD affect the oral health status of patients by inducing gingival hyperplasia, xerostomia, calcification of root canals and delayed eruption of teeth. Periodontitis increases systemic inflammatory burden leading to worsening of CKD which in turn has been has been found to negatively affect CKD of patients on hemodialysis therapy by altering their serum albumin and C-reactive protein levels. As hypoalbuminemia leads to increased mortality in CKD patients, it needs to be avoided by reducing systemic inflammatory burden in patients receiving HD therapy. Treating periodontal disease could be one factor that might decrease the systemic inflammatory burden and thereby improve quality of life of these patients.

***Sources of Data: ***Data from descriptive, cross sectional and longitudinal studies published between 2000 and 2012 were included. Data searches based on human studies only.

***Data Extraction: ***The key words, periodontitis, chronic kidney disease and hemodialysis, on MEDLINE, approximately 120 studies were identified. 35 of them were relevant to all three keywords. Most of them were cross sectional studies and total 7 clinical trials were identified regarding checking of serum levels after periodontal therapy with variable results.

***Conclusion:*** Patients with CKD have higher prevalence of periodontal disease while non-surgical periodontal therapy has been indicated to decrease the systemic inflammatory burden in patients with CKD specially those undergoing HD therapy.

## OVERVIEW

 Non communicable diseases (NCDs) are chronic medical diseases or conditions which do not spread by infections. Common NCDs are cardiovascular diseases (CVD), diabetes mellitus (DM), pulmonary diseases, osteoporosis and chronic kidney diseases (CKD).^1^ According to the WHO definition, oral diseases were included in NCDs in 2005 as these are source of infection to other organs of the body.^2^ The effects of NCDs are in terms of mortality, morbidity and financial crisis. It is predicted by a global survey that by 2020, 7 out of every 10 deaths will be due to NCDs in the developing countries.^3^ According to a report, 80% of morbidities due to CVDs and DM and 40 % of total deaths due to cancers can be controlled by eliminating the risk factors.^4^

***Risk Factors: ***The main risk factors involved in NCDs are obesity, decreased physical activities, raised blood pressure and increased blood cholesterol levels.^4^ An important potential risk factor for NCDs highlighted recently is periodontal disease. It is destructive chronic infection of the gums, ligaments, and bone, predominantly caused by Gram-negative bacteria residing in dental plaque biofilm.^5^ Researchers reported PD as an increased risk for various chronic diseases. It contributes to systemic diseases due to increased inflammatory burden which leads to worsening of CKD.^6^ Pathogens cause destruction of periodontal tissues resulting in detachment of tissues from around the teeth and entry of pathogens and their products in systemic circulation leading to increased systemic inflammation.^7^ The association of periodontal disease with DM and CVD is well documented but its contribution in CKD is still debatable.

***Bidirectional Relationship between Systemic diseases and Oral diseases: ***American dental association highlighted 200 possible connections between systemic diseases and oral health.^8^ Studies have established a relationship of systemic conditions like CVD, DM, pulmonary diseases, osteoporosis, anemia and CKD with oral diseases but the relationship yet established is an association and not a causation.^1^ In some situations a bidirectional model has also been observed. Presence of one condition increases the chances of others. On the other hand controlling one condition may benefit the patient regarding the other condition.

 The effects of systemic diseases on oral environment are well documented in many ways. Impaired immune system, abnormal collagen formation and inflammatory burden in patients with diabetes effect oral tissues.^9^ Pathogenic bacteria from oral environment may activate an inflammatory cascade in patients with diabetes.^9^ In Cardiovascular diseases (CVD), raised serum level of pro-inflammatory markers increases the chances of gingival and periodontal diseases as evident from gingival biopsies of CVD patients.^10^ In pulmonary diseases, chances of fungal infection in oral cavity are increased due to inhaler usage.^11^ In the recent past, research work has been carried our regarding effects of CKD on oral tissues and the effect of oral diseases on worsening the prognosis of patients with End Stage Renal Disease (ESRD).

***End Stage Renal Disease (ESRD)*** *:* It is a systemic condition which significantly affects oral hard and soft tissues. One of the main effects is enamel hypoplasia due to disturbance in enamel formation and mineralization.^12^ Other manifestations of chronic kidney disease and hemodialysis (HD) therapy are xerostomia, enamel hypoplasia, calcification of root canals, abnormal pH of saliva and abnormal delay in eruption of teeth.^12^ Community studies in Pakistan show a high percentage of chronic kidney diseases in the general population.^13^

***Effect of CKD on Periodontitis: ***Studies revealed that CKD affects teeth, oral mucosa, periodontium, salivary glands, and tongue resulting in a negative effect on the oral health status of the patient.^14^ Many cross sectional studies as well as clinical trials have been carried out regarding this aspect. Increased levels of plaque have been reported for hemodialysis (HD) populations from several countries including Brazil.^15^ The poor oral hygiene and increased level of plaque and gingival inflammation have been attributed to neglected oral care due to presence of ESRD.^16^

***Extent and Severity of Periodontitis:*** Regarding the extent and severity of periodontitis among CKD patients, conflicting reports are present in literature. Some studies showed higher prevalence of periodontal disease in CKD patients^15^ while others did not show any significant difference in prevalence of PD between CKD and non CKD patients.^17^ Seven cross sectional studies conducted in Brazil, Canada, Turkey, USA and Taiwan reported that chronic severe periodontitis was significantly more frequent among HD patients as compared to normal persons and periodontal disease was comparatively more severe and prevalent in CKD patients.^18^^-^^22^ These studies enrolled above 1000 study subjects for a better comparison among patients and healthy controls. Based on Community periodontal Index of Treatment needs (CPITN), Borawski et al. (2007) also presented high severity of periodontitis as compared to healthy population.^23^ Using attachment loss as an indicator of periodontitis, Thorman et al. (2009) reported that HD patients had significantly more attachment loss as compared to healthy individuals.^24^ Studies focusing on the periodontal health of End Stage Renal disease (ESRD) patients on HD maintenance therapy have reported the presence of poor oral hygiene and gingival inflammation in study subjects.^22^

 However cross sectional studies from Spain and Netherland reported that they did not find any significant association between periodontal disease and CKD in HD patients.^25^ They enrolled 105 patients on HD therapy and compared them with healthy population. Results were not statistically significant regarding association of CKD with periodontal disease. These studies are from developed countries and these results are may be due to the fact that dental treatment is a part of their routine therapy. It is important to mention that studies reporting higher prevalence examined a larger number of patients as compared to other studies.

***Clinical Trials:*** Clinical trials regarding this topic also showed different results. In a clinical trial performed on 352 patients, researcher found an increased severity of periodontitis in HD patients as compared to normal healthy persons.^21^

 On the other hand, Bots CP et al (2006) in a study from Netherlands of End Stage Renal Disease (ESRD) patients, some of whom were receiving HD, did not find an increased loss of attachment when compared with some healthy case-matched controls. Periodontal status of ESRD patients receiving HD showed no increase in periodontal indices when compared with case-matched controls.^26^ The authors noticed that the HD group had greater numbers of periodontopathic bacterial species than the control group.^26^ After adjusting other risk factors, periodontitis was highlighted as an independent risk factor for CKD in most of the trials.^23^

**Effect of Periodontitis on CKD:**

***Pathogenesis*** *:* A proposed mechanism for the effect of periodontitis on the development of kidney disease is systemic inflammation.^27^ Periodontal pathogens have been shown to have the ability to adhere to, invade, and proliferate in coronary endothelial cells leading to atheroma formation and impaired vasculature relaxation. Cardiovascular diseases and CKD share many risk factors, so it can be assumed that periodontal disease exerts similar effects within the vasculature of the kidney.^28^ Both periodontitis and kidney diseases are associated with inflammatory markers such as C-reactive protein and chronic low level inflammation associated with periodontitis may lead to endothelial dysfunction which plays a role in the pathogenesis of kidney disease in edentulous patients.^29^ The deleterious effects of systemic inflammation on kidney function could occur during the period of active periodontal infection and accumulate during the life time of the individual. Inflammation is an important predictor of low serum albumin levels among dialysis patients.^30^

***Serum Markers:*** Regarding the serum markers of inflammation in HD patients, serum markers related to periodontitis that have been studied include albumin and CRP.^6^ Researchers have reported that hemodialysis (HD) patients have elevated levels of C-reactive protein (CRP) as compared to normal conditions and periodontal disease is associated with an elevation of serum levels of CRP.^31^ CRP is a vague indicator of inflammation which is raised in various infections, workers of industrial zone and some other conditions.^32^ It cannot be taken as specific indicator of inflammation in CKD patients on HD therapy as it may be raised due to some other condition rather than CKD. However albumin level is a specific indicator of prognosis of CKD and quality of hemodialysis.^15^

***Albumin:*** Hypoalbuminemia has been demonstrated to be a strong predictor of death in chronic renal failure.^14^ Chronic inflammation appears to be the culprit in most of the patients with low levels of albumin. Some studies have suggested that hypoalbuminemia may be more indicative of underlying inflammation, rather than nutritional status, especially in patients with kidney disease.^6^ In a study it was observed that on dialysis initiation in the US pediatric population, hypoalbumenimic patients were at higher risk of dying as compared to patients in whom dialysis was initiated with normal albumin levels.^33^ It was also assessed that hypoalbuminemia is highly prevalent in kidney failure and is associated with an increased mortality risk in this population.^15^ In severe periodontal disease increasing systemic inflammation can lead to hypoalbuminemia in CKD patients thus worsening the prognosis of patients of ESRD undergoing HD therapy.^15^

 In a case control study, Kshirsagar AV et al. (2007) observed an association of severe periodontal disease and hypoalbuminemia in a group of patients who were receiving long-term outpatient hemodialysis.^15^ In study subjects, patients with periodontal disease were three times more likely to have low serum albumin than patients without periodontal disease.^15^

***Clinical Trials: ***Using the key words, periodontitis, chronic kidney disease and hemodialysis, on MEDLINE, approximately 120 studies were identified. Thirty five of them were relevant to all three keywords. Most of them were cross sectional studies and total 7 clinical trials were identified regarding checking of serum levels after periodontal therapy with variable results (Table-I). Some clinical trials showed that periodontal treatment brings changes in serum inflammatory markers of CKD patients and successful periodontal therapy decreases serum CRP levels, IL-6 and LDL-cholesterol.^34^

**Table-I:** Clinical Trials regarding Effect of periodontal therapy in CKD patients.

*Researchers (Location)*	*Participants*	*Outcome measures*	*Results*
D'AiutoF, et al. (London)	65	Serum inflammatory markers CRP, IL-6	Reduction after treatment
Mattila K, et al. (Finland)	35	CRP, fibrinogen	CRP decreased Fibrinogen no effect
MercanogluF, et al. (Turkey)	54	Endothelial dysfunction	Improvement after periodontal treatment
Kadiroglu AK, et al. (Turkey)	41	CRP, ESR, Hb	CRP and ESR decreased and Hb increased after periodontal treatment
Artese HP, et al. (Brazil)	40	GFR	Significant improvement after therapy
Radafshar G, et al. (Iran)	35	CRP	Significant decrease after therapy
Vilela EM, et al. (Brazil)	56	CRP, IL-6, Prohepcidin	CRP decreased, IL-6 decreased, Prohepcidin levels decreased

CRP: C-Reactive Proteins, IL-6: Interleukin-6, ESR: Erythrocyte Sedimentation Rate,

Hb: Hemoglobin, GFR: Glomerular Filtration Rate

 An impressive 3-fold decrease in C-reactive protein and a rise in hemoglobin (Hb) levels in HD patients were reported to occur already after 4–6 weeks following traditional periodontal therapy.^35^ D’Aiuto et al. (2004) reported decrease in CRP levels after periodontal therapy^36^ while some researchers reported no significant change in serum markers.^37^ Another interventional study demonstrated that periodontal therapy has a positive effect on Glomerular Filtration Rate (GFR) of CKD patients.^27^ Vilela et al (2011) reported the findings of a randomized controlled trial that periodontal therapy decreases level of serum prohepcidin in chronic kidney disease patients.^38^

 The issue of poor oral health status in CKD patients apparently deserves a higher awareness of the problem, and increased attention, and indicates the need for a closer collaboration between primary care physicians, nephrologists and dentists.^23^

## Conclusions

 Literature supports a bidirectional relation between CKD and periodontal disease. Patients with CKD have higher prevalence of periodontal disease while non-surgical periodontal therapy has been indicated to decrease the systemic inflammatory burden in patients with CKD specially those undergoing HD therapy. Most of the trials conducted have observed the effect of periodontal therapy on CRP levels while none of them used albumin levels as outcome measure after periodontal therapy. As albumin is a strong prognostic marker in ESRD patients on HD therapy, it needs to be as an outcome measure in clinical trials evaluating the effect of periodontal treatment on impairing quality of life of CKD patients.
